# The Enterocyte-Associated Intestinal Microbiota of Breast-Fed Infants and Adults Responds Differently to a TNF-α-Mediated Pro-Inflammatory Stimulus

**DOI:** 10.1371/journal.pone.0081762

**Published:** 2013-11-26

**Authors:** Manuela Centanni, Silvia Turroni, Clarissa Consolandi, Simone Rampelli, Clelia Peano, Marco Severgnini, Elena Biagi, Giada Caredda, Gianluca De Bellis, Patrizia Brigidi, Marco Candela

**Affiliations:** 1 Department of Pharmacy and Biotechnology, University of Bologna, Bologna, Italy; 2 Institute of Biomedical Technologies - Italian National Research Council, Milan, Italy; Charité, Campus Benjamin Franklin, Germany

## Abstract

Co-evolved as an integral component of our immune system, the gut microbiota provides specific immunological services at different ages, supporting the immune education during our infancy and sustaining a well-balanced immunological homeostasis during the course of our life. In order to figure out whether this involves differences in the microbial groups primarily interacting with the host immune system, we developed a non-invasive HT29 cell-based minimal model to fingerprint the enterocyte-associated microbiota fraction in infants and adults. After depicting the fecal microbial community of 12 breast-fed infants and 6 adults by 16S rDNA amplicon pools 454 pyrosequencing, their respective HT29 cell-associated gut microbiota fractions were characterized by the universal phylogenetic array platform HTF-Microbi.Array, both in the presence and absence of a tumor necrosis factor-alpha (TNF-α)-mediated pro-inflammatory stimulus. Our data revealed remarkable differences between the enterocyte-associated microbiota fractions in breast-fed infants and adults, being dominated by *Bifidobacterium* and *Enterobacteriaceae* the first and *Bacteroides-Prevotella* and *Clostridium* clusters IV and XIVa the second. While in adults TNF-α resulted in a profound impairment of the structure of the enterocyte-associated microbiota fraction, in infants it remained unaffected. Differently from the adult-type gut microbial community, the infant-type microbiota is structured to cope with inflammation, being co-evolved to prime the early immune response by means of transient inflammatory signals from gut microorganisms.

## Introduction

It is a matter of fact that the human genome does not code for sufficient information to carry out all functions necessary to maintain health. Indeed, for several aspects of our physiology, such as nutrition [1], protection from pathogens [2] and immunological wellbeing [3,4], we strictly depend on our symbiont microbial partner, the gut microbiota (GM). Being extremely dynamic and rapidly adaptable, the gut microbiome represents a plastic coding entity of the human superorganism, strategic to preserve health and homeostasis through the entire life course [5]. This GM plasticity allows our microbial counterpart to adjust the ecological services [6] in response to the specific host needs at different ages [7-10]. In particular, evolved as an integral component of the immune system, the human GM finely calibrates the immunological services at the different host ages [11], supporting the process of immune education during our infancy and maintaining a balanced immune homeostasis along the adult life.

Breast-fed infants possess a peculiar GM structure which is dominated by *Bifidobacterium* and *Enterobacteriaceae* [12,13]. Within a critical “time window” of 8 months of life [14], the infant-type GM plays specific functions strategic for the correct maturation of the host immune system functionalities [11], modulating the T cell differentiation process [15] and leading to the acquisition of the mucosal iNKT cell tolerance [16]. These findings have been strengthened by two recent perspective surveys of GM in Danish and Swedish infants, which provided robust evidences that a low bacterial diversity in the early life is associated with an increased risk of immunological disorders later in life [17,18]. Further, the neonatal intestinal immune apparatus has been recently reported as highly responsive to microbial ligands [19], being primed to establish an intense microbe-host immunological cross-talk since birth [11]. At weaning, with the introduction of solid foods, the GM progressively acquires an adult-like profile which is dominated by *Bacteroidetes* and *Firmicutes* [20]. This phylogenetic and functional GM architecture provides different immunological functions, specifically calibrated on the needs of the adult host. Indeed, the adult-type microbiota has been reported as essential to maintain a state of alert of the adult innate and adaptive immune system [21] and, at the same time, to preserve the immunological homeostasis favoring a constitutive low-grade physiological inflammatory status [3]. 

Even if several steps forward have recently been made in the comprehension of the developmental trajectory of the GM-host immunological cross-talk from early infancy to adulthood, the great majority of the studies have been carried out in stool samples without providing information on the specific structure of the mucosa-associated microbiota [22]. However, bacteria interacting with the gut mucosal surface have a role of primary importance in the cross-talk with the host immune system [21]. Establishing a close interaction with the epithelial apex, mucosal microorganisms enhance the level of epithelial cross-talk at the enterocyte surface, shaping the gut immunological environment [23].

In order to shed light on the functional specificity of the GM-host immunological interaction in infants and adults – and being aware of the impossibility of invasive sampling in healthy infants – in the present paper we developed a non-invasive HT29 cell-based minimal model to characterize the enterocyte adherent GM fraction in human beings. In the light of the fact that stools are considered as representative of the mucosa-associated GM [24,25], our approach involved the co-incubation of freshly produced and immediately processed fecal samples with monolayers of the human enterocyte line HT29 [26]. The enterocyte-associated GM fraction was subsequently characterized by a dual approach based on qPCR and the phylogenetic universal array platform HTF-Microbi.Array [27,28]. 

 By using our *ex vivo* HT29 cell-based model, we investigated the phylogenetic structure of the enterocyte-associated microbiota fraction of 12 breast-fed infants and 6 young adults, whose GM composition was characterized by pyrosequencing of the 16S rDNA V4 region. In order to mimic a host inflammatory response, experiments were performed in the presence or absence of a tumor necrosis factor-alpha (TNF-α)-mediated inflammatory stimulus. This pro-inflammatory cytokine was selected because pivotal in the intestinal inflammatory processes [29]. According to our data, breast-fed infants and adults possess phylogenetically and functionally different enterocyte-associated GM fractions. While in adults the enterocyte adherent fraction is significantly distorted by the inflammatory stimulus, in breast-fed infants the enterocyte-associated microbiota remains unchanged in the inflammatory context, showing a phylogenetic and functional structure that we hypothesize is co-evolved to cope with transient inflammatory responses. 

## Materials and Methods

### Subject enrolment and sample collection

Twelve exclusively breast-fed genetically unrelated infants (age: 2-8 months) and 6 adults (age: 30-40 years) were recruited for this study in the Bologna metropolitan area and surroundings, Italy (Table S1). All subjects were healthy and had not received antibiotics or probiotics or prebiotics for at least 3 months prior to the sampling. The study protocol was approved by the Ethics Committee of S. Orsola-Malpighi University Hospital (Bologna, Italy). Written informed consent was obtained from the 6 adults as well as from both parents of each infant. All participants were asked to collect one fecal sample, store it at 4°C and bring it to the research laboratory within 24 h. Stool samples from infants were collected by parents. After collection, fecal samples were immediately processed.

### Fecal slurry preparation

Fecal slurries were prepared as reported by Centanni et al. [28]. Briefly, stools were immediately diluted 1:2 in ice-cold Dulbecco’s modified Eagle’s medium (DMEM; Sigma-Aldrich, St. Louis, MO) and homogenized in a Stomacher blender (VWR International PBI, Milan, Italy) for 2 min at high speed, until uniform consistency was achieved. For each subject, a 1/100 dilution of the fecal slurry containing approximately 10^10^ bacterial cells was prepared in DMEM for the interaction with the human colonic epithelial cell line HT29.

### HT29 cell culture conditions and evaluation of MUC2 transcriptional levels

HT29 cell line was grown in DMEM with 4.5 g/l glucose supplemented with 10% heat-inactivated foetal bovine serum (FBS; Sigma-Aldrich), 1% L-glutamine (Sigma-Aldrich) and 1% penicillin-streptomycin (Sigma-Aldrich), as reported by O’Hara et al. [30]. Cells were routinely propagated in 75-cm^2^ flasks (BD Falcon; Becton Dickinson, Heidelberg, Germany) at 37°C and 5% CO_2_ in a humidified atmosphere until they reached 90% confluence. For fecal slurry-HT29 cell interaction assays, 2.5 x 10^5^ HT29 cells were seeded per well in 24-well tissue culture plates (BD Falcon), and allowed to grow to confluent monolayers. For the visualization of HT29 cell adherent fecal bacteria, 2.5 x 10^5^ HT29 cells were layered on 12 mm-diameter glass coverslips in 24-well tissue culture plates (BD Falcon) and grown until confluence. Twenty-four hours before the assays, the cell medium was replaced with interaction medium (DMEM, 25 mM HEPES, 1 g/l glucose [Gibco BRL, Life Technologies, Grand Island, NY], 1% FBS) with or without the addition of 2 ng/ml human recombinant TNF-α (Thermo Scientific, Milan, Italy) [30,31]. According to Porath et al. [32], the stimulation of HT29 cells with TNF-α for 24 h induces a substantial increase in mRNA levels of several cytokines and chemokines, such as TNF-α, interleukin (IL)-8, macrophage inflammatory protein (MIP)-2, MIP-3α, growth-regulated oncogene (GRO)-α, GRO-γ and interferon-inducible protein-10 as well as COX-2.

In control experiments, the mucus production by HT29 cells was evaluated both in the presence and absence of TNF-α. To this aim, the transcriptional levels of the intestinal mucin gene MUC2 were determined as reported by Dolan et al. [33] and compared. Briefly, total RNA was extracted from HT29 cells pre-treated or not with TNF-α, with the Illustra RNAspin Mini Kit (GE Healthcare, Milan, Italy), and single-stranded cDNA was synthesized using random hexamers and the High Capacity cDNA Reverse Transcription Kit (Applied Biosystems, Foster City, CA). qPCR was carried out with a LightCycler SYBR Green system (Roche, Mannheim, Germany), using primers and conditions described by Dolan et al. [33]. Beta actin was used as a reference gene [34,35]. Relative mRNA expression of MUC2 was determined using the ΔΔCT method after Pfaffl correction [36]. According to our data, MUC2 expression was observed at basal levels in control cells and not significantly affected by TNF-α stimulation (data not shown).

### Fecal slurry-HT29 cell interaction assay

Before interaction assays, interaction medium was collected from HT29 cell monolayers and replaced with 1 ml of a 1/100 dilution of freshly prepared fecal slurry. Cells were next incubated for 1 h at 37°C and 5% CO_2_ in a humidified atmosphere, as reported for standard bacterial adhesion assays [37]. After 3 washings with PBS, 200 µl of 0.05% trypsin/0.02% EDTA (Sigma-Aldrich) were added to each well and incubated for 10 min at 37°C to detach cells and adherent bacteria [38]. Wells were rinsed with 200 µl of PBS and samples were stored at -20°C for subsequent analysis. As a control, 1 ml of 1/100 dilutions of fecal slurries was incubated with or without 2 ng/ml TNF-α for 1 h at 37°C and 5% CO_2_ in a humidified atmosphere.

### Visualization of the HT29 cell-associated microbiota fraction by fluorescence microscopy

Bacterial cells were stained as reported by Vesterlund et al. [39]. Briefly, 1 ml aliquots of 1/100 dilutions of fecal slurries were incubated with 0.2 µg/ml DAPI (Sigma-Aldrich) at room temperature in the dark for 30 min with mild shaking. After washing, stained slurry dilutions were resuspended in 500 µl of interaction medium and added to HT29 cell monolayers previously grown on glass coverslips. After 1-h incubation at 37°C and 5% CO_2_ [37], wells were rinsed with PBS to remove non-adherent bacteria, and HT29 cells were fixed on coverslips with 1% paraformaldehyde at 4°C for 1 h. Glass coverslips were mounted in Fluoromount-G (SouthernBiotech, Birmingham, AL) and incubated 1 h at room temperature before fluorescence microscopy observation (ECLIPSE 90i, Nikon, Melville, NY). The Nis-Elements AR 3.2 software (Nikon) was used for image acquisition. For each sample, pictures of the same area were taken at 100x magnification under both fluorescent light and phase contrast. For each coverslip, 5 different microscopic views were taken. Three independent adhesion experiments were performed for each experimental condition.

### Microbial DNA extraction from fecal slurry and enterocyte-associated microbiota fraction

Total microbial DNA from fecal slurries was extracted by using QIAamp DNA Stool Mini Kit (QIAGEN, Hilden, Germany) according to the protocol reported by Candela et al. [27].

Genomic DNA extraction from the enterocyte-associated microbiota fraction was carried out using DNeasy Blood & Tissue Kit (QIAGEN) with a modified protocol. Briefly, cell pellets were suspended in enzymatic lysis buffer (20 mM Tris HCl, pH 8.0, 2 mM EDTA, 1.2% Triton X-100, 20 mg/ml lysozyme) and incubated at 37°C for 30 min. After adding 200 µl of AL buffer (QIAGEN), one 3-mm glass bead and 0.15 g of 0.1-mm zirconia beads, samples were beaten in a FastPrep-24 instrument (MP Biomedical, Irvine, CA) at 5.5 m/s for 2 min, and further processed according to the manufacturer’s instructions.

DNA concentration and quality were evaluated using NanoDrop ND-1000 (NanoDrop Technologies, Wilmington, DE).

### 16S rDNA gene amplification

For the amplification of the V4 region of the 16S rDNA gene the primer set 520F (5’-AYTGGGYDTAAAGNG-3’) and 802R (5’-TACNVGGGTATCTAATCC-3’) [40] was utilized. These primers were designed to include at their 5’ end one of the two adaptor sequences used in the 454-sequencing library preparation protocol (adaptor A and B), linked to a unique MID tag barcode of 10 bases allowing the identification of the different samples. PCR mixtures contained 0.5 µM of each forward and reverse primer, 100 ng of template DNA, 2.5 U of GoTaq Flexi Polymerase (Promega, Milan, Italy), 200 µM of dNTPs and 2 mM of MgCl_2_ in a final volume of 50 µL. Thermal cycling consisted of an initial denaturation step at 95°C for 5 min, followed by 35 cycles of denaturation at 94°C for 50 s, annealing at 40°C for 30 s, and extension at 72°C for 60 s, with a final extension step at 72°C for 5 min [40]. PCR amplifications were carried out in a Biometra Thermal Cycler T Gradient (Biometra, Göttingen, Germany). 

### Pyrosequencing of fecal slurries

The PCR products derived from amplification of the specific 16S rDNA V4 hypervariable region were individually purified with MinElute PCR Purification Kit (QIAGEN) and then quantified using the Quant-iT PicoGreen dsDNA kit (Invitrogen, Leek, Netherlands). After the individual quantification step, amplicons were pooled in equal amounts (thus creating a 10-plex and an 8-plex pool) and one more time purified by 454-Roche Double Ampure size selection protocol with Agencourt AMPure XP DNA purification beads (Beckman Coulter Genomics GmbH, Bernried, Germany), in order to remove primer dimers, according to the manufacturer’s instructions (454 LifeSciences, Roche, Branford, CT).

Amplicon pools were fixed to microbeads to be clonally amplified by performing an emulsion PCR following the GS-FLX protocol Titanium emPCR LIB-A (454 LifeSciences, Roche). Following this amplification step, the beads were enriched in order to keep only those carrying identical PCR products on their surface, and then loaded onto a picotiter plate for pyrosequencing reactions, according to the GS-FLX Titanium sequencing protocol. The two pools were sequenced in one eight of a plate each. Amplicon sequences were deposited in MG-RAST under the project ID 5838. 

### Bioinformatic analysis of 16S rDNA gene sequencing data

Sequencing reads were analyzed using the QIIME pipeline [41], as described in Claesson et al. [42]. Briefly, V4 sequences were filtered according to the following criteria: (i) exact matches to primer and barcode sequences; (ii) read length not shorter than 150 bp and not longer than 350 bp; (iii) no ambiguous bases (Ns); (iv) a minimum average quality score over a 50-bp rolling window of 25. For bacterial taxonomy assignment we utilized RDP-classifier (version 2.2) with 50% as confidence value threshold. Trimmed reads were clustered into OTUs at 97% identity level and further filtered for chimeric sequences using ChimeraSlayer (http://microbiomeutil.sourceforge.net/#A_CS). Alpha-diversity and rarefaction plots were computed using four different metrics: Shannon, PD whole tree, chao1 and observed species. Weighted and unweighted UniFrac distances were used to perform Principal Coordinates Analysis (PCoA) and Procrustes superimposition in order to evaluate β-diversity and the correlation between pyrosequencing and phylogenetic microarray data. PCoA, Procrustes, heatmap and bar plots were built using the packages Made4 [43] and Vegan [44] in R 3.0.0. The R packages Stats and Vegan were used to perform statistical analysis. In particular, Wilcoxon signed-rank test was used to compare infant and adult gut microbiota for α- and β-diversity; data separation in the PCoA was tested using a permutation test with pseudo F-ratios (function adonis in the Vegan package); Fisher’s exact test was used to assess the significance of differences between clusters from the hierarchical clustering analysis; Student’s t-test or Mann-Whitney U test were used to carry out significant differences at phylum, group or genus level. When appropriate, *P* values were adjusted for multiple comparison using the Benjamini-Hochberg correction. False discovery rate (FDR) < 0.05 was considered as statistically significant. The function protest was utilized to validate the Procrustes analysis. A P value < 0.05 was considered as statistically significant.

### HTF-Microbi.Array/qPCR combined approach

In order to characterize the phylogenetic structure of fecal slurries and enterocyte-associated GM fractions, the HTF-Microbi.Array/qPCR combined approach developed by Centanni et al. [28] was employed. This approach is based on the association of the High Taxonomic Fingerprint (HTF)-Microbi.Array [27] - a fully validated phylogenetic microarray platform which allows the detection and quantification of up to 31 intestinal bacterial groups, covering up to 95% of the human GM – with a qPCR protocol designed to specifically quantify *Bifidobacterium* spp. In brief, for each sample the non-bifidobacterial fraction was characterized using the HTF-Microbi.Array, whereas the relative abundance of *Bifidobacterium* was estimated by qPCR. Microarray platform description and fluorescence raw data were uploaded in GEO archive under the ID GSE51177.

## Results

### 16S rDNA sequencing of the fecal microbiota in breast-fed infants and adults and validation of the HTF-Microbi.Array for the high taxonomic level fingerprinting of the human fecal microbiota

**Figure 1:**
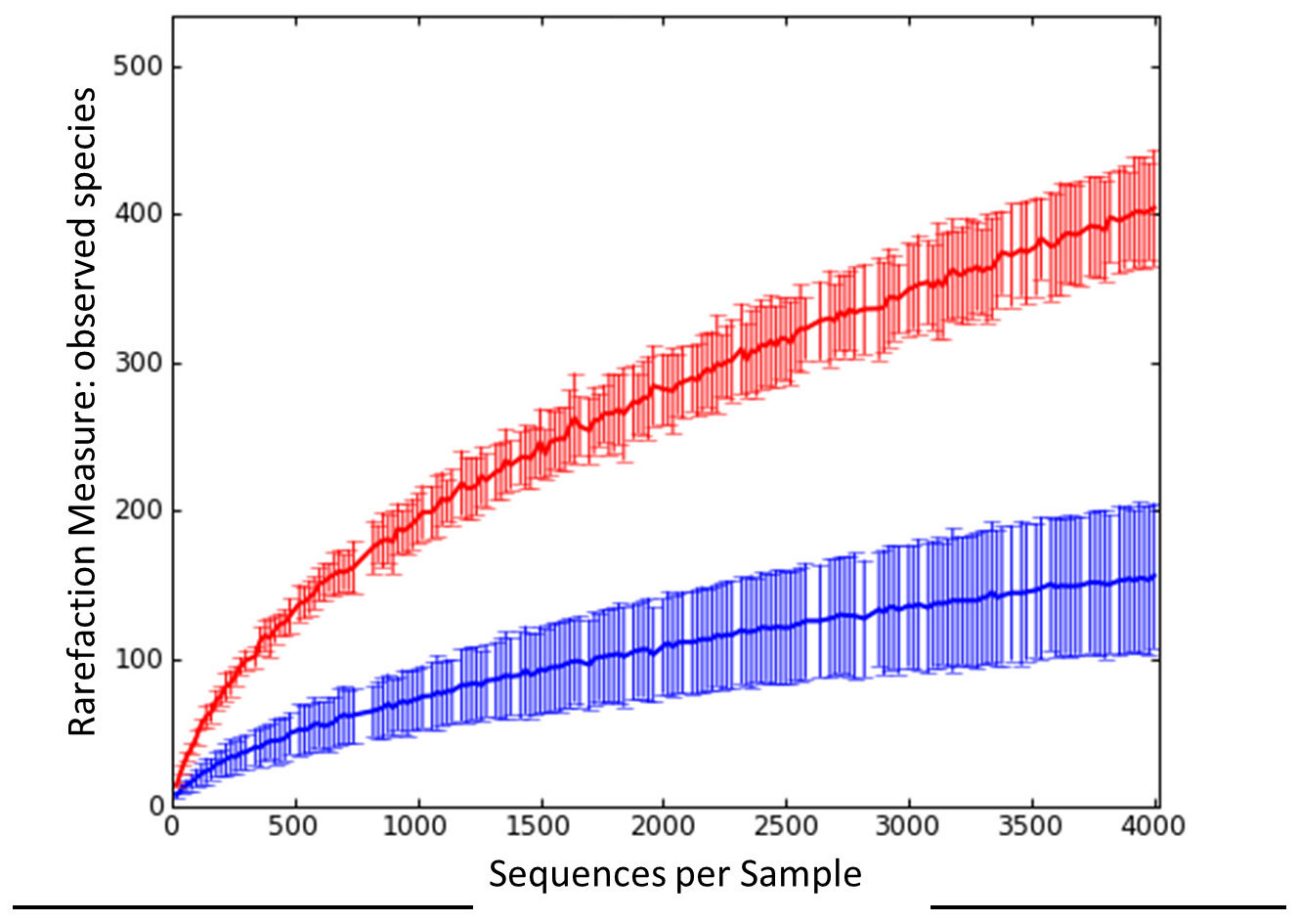
Rarefaction curves generated for 16S rDNA gene sequences from stool samples of breast-fed infants and adults. Curves show the observed species in the fecal microbial communities of breast-fed infants (blue, n=12) and adults (red, n=6).

**Figure 2:**
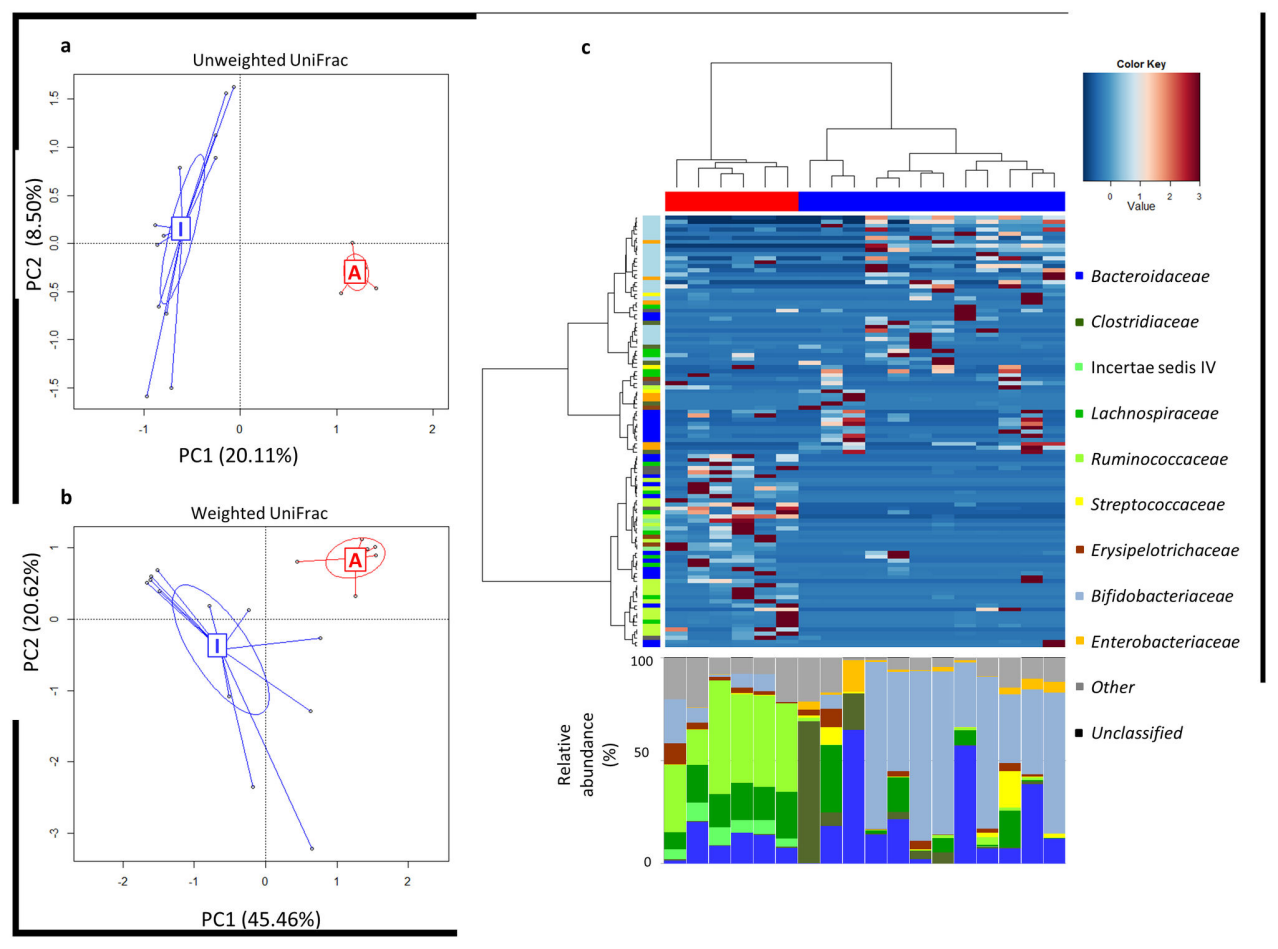
Analysis of 16S rDNA gene sequences from stool microbiota separates breast-fed infants and adults. Unweighted (a) and weighted (b) UniFrac PCoA of the fecal microbiota from breast-fed infants (blue) and adults (red). c, Hierarchical Ward-linkage clustering based on the Spearman correlation coefficients of OTU proportion. OTUs were filtered for subject prevalence of at least 20%. Subjects are clustered in the top of panel and color-coded as in a. OTUs are clustered by the vertical tree and color-coded by family assignment. 107 OTUs confidentially classified to family level are visualized. In the bottom panel the relative abundance (%) of family-classified microbiota is shown.

The fecal microbial communities of 12 breast-fed infants and 6 adults were phylogenetically characterized using 16S rDNA gene pyrosequencing (Figure S1). A total of 127,530 high-quality sequence reads from the 16S rDNA V4 region were obtained, with an average of 7,085 reads per subject. Reads were clustered at 97% identity in 3,471 OTUs. Rarefaction curves indicated a lower diversity in the fecal microbial community of infants with respect to adults (Figure 1). Comparison of community richness using several metrics showed a significantly (*P* < 0.001) lower degree of α-diversity in the infant fecal microbiota with respect to the adult one (Shannon: infants, 3.16 ± 0.91 vs adults, 6.00 ± 0.25; PD whole tree: 22.02 ± 7.42 vs 48.00 ± 5.18; chao1: 294.26 ± 108.62 vs 807.68 ± 130.32; observed species: 156.50 ± 50.56 vs 400.67 ± 44.07). PCoA analysis of the unweighted (Figure 2a) and weighted (Figure 2b) UniFrac distances indicated a sharp (*P* < 0.001) separation between the infant and adult fecal microbial communities, with a significantly (*P* < 0.001) higher degree of interindividual variability within breast-fed infants. In particular, for breast-fed infants mean values of unweighted and weighted UniFrac distances of 0.758 ± 0.045 and 0.843 ± 0.321 were obtained, respectively, whereas for adults corresponding values of 0.693 ± 0.014 and 0.528 ± 0.153 were obtained. Moreover, clustering analysis of the OTU-based community structures resulted in a significant (*P* < 0.001) separation of infants from adults (Figure 2c). While the adult GM was dominated by *Bacteroidaceae* (mean relative abundance, rel. ab. ± SEM, 11% ± 3%), *Clostridiales* Incertae Sedis IV (13% ± 1%), *Lachnospiraceae* (17% ± 2%) and *Ruminococcaceae* (39% ± 5%), with *Veillonellaceae* (1% ± 0.7%), *Erysipelotrichaceae* (3% ± 1%) and *Bifidobacteriaceae* (7% ± 3%) as subdominant components, the infant one was largely dominated by *Bifidobacteriaceae* (45% ± 9%), with *Bacteroidaceae* (20% ± 6%) as secondary dominant component. *Clostridiaceae* (9% ± 6%), *Lachnospiraceae* (7% ± 3%) and *Enterobacteriaceae* (4% ± 1%) were subdominant groups, whereas *Enterococcaceae* (1% ± 0.6%), *Streptococcaceae* (3% ± 1%) and *Erysipelotrichaceae* (2% ± 0.8%) represented minor components of the fecal microbial community of breast-fed infants. In Table 1 the significant differences at phylum and genus level between the fecal microbiota composition of breast-fed infants and adults are reported.

**Table 1:** Bacterial phyla and genera showing a significantly different relative abundance in the fecal microbiota of breast-fed infants and adults.

	**Relative abundance** (**mean, %**)		
**Phylum**	**Breast-fed infants**	**Adults**	*P value
***Actinobacteria***	46.0	7.6	0.024
***Firmicutes***	28.7	76.3	0.007
***Proteobacteria***	3.5	0.4	0.003
**Genus**			
***Bifidobacterium***	45.4	7.5	0.049
***Alistipes***	0	1.3	<0.001
***Streptococcus***	2.8	0.2	0.007
***Blautia***	0.04	6.6	<0.001
***Lachnospiraceae*; Unclassified**	2.1	5.7	0.041
***Roseburia***	0.03	6.8	<0.001
***Faecalibacterium***	0.4	19.8	<0.001
***Oscillibacter***	0.01	1.5	<0.001
***Ruminococcaceae*; Unclassified**	0.3	3.8	0.002
***Ruminococcus***	0.03	8.7	0.011
***Subdoligranulum***	0.1	3.0	0.001
***Veillonella***	1.7	0.01	0.006
***Erysipelotrichaceae*; Unclassified**	0.3	2.8	0.004
***Escherichia/Shigella***	2.6	0.06	0.001

* Mann-Whitney U-test.

The comparison between the phylogenetic structure of the fecal microbial communities of breast-fed infants and adults revealed profound differences, reflecting an overall different architecture of the infant and adult gut microbial ecosystem at both phylum and family level. We previously developed a combined approach based on HTF-Microbi.Array and qPCR for the high taxonomic level fingerprint of the human gut microbiota [28]. In order to further validate this approach, we compared with the 16S rDNA V4 pyrosequencing in providing the high taxonomic level fingerprint of the fecal microbial community of the 12 breast-fed infants and the 6 adults enrolled in our study. Procrustes similarity analysis of the weighted UniFrac distances obtained by 16S rDNA pyrosequencing and the Euclidean distances based on rel. ab. of the HTF-Microbi.Array/qPCR profiles revealed a significant relationship between the two datasets (*P* < 0.001) (Figure S2). These data supported the overall concordance of the two approaches in depicting the high taxonomic level fingerprint of the human gut microbiota, confirming the reliability of the HTF-Microbi.Array/qPCR combined approach. 

### The HT29 cell-associated microbiota fraction in breast-fed infants and adults

In order to approximate the human intestinal mucosa-associated GM fraction we developed a non-invasive *ex vivo* approach based on the human enterocyte line HT29. Briefly, stools were processed within 24 h after collection to obtain a homogeneous microbiota community suspension. Bacterial cells were next incubated with monolayers of HT29 cells as performed in standard bacterial adhesion assays [37]. After washing, the enterocyte-associated GM fraction was characterized by the previously validated HTF-Microbi.Array/qPCR dual approach [28]. *Ex vivo* HT29 cell-based experiments were carried out for the 12 breast-fed infants and the 6 adults enrolled in our study (Figure S3). As a control, the presence of fecal bacterial cells on the HT29 cell surface was determined by DAPI fluorescence staining. Clusters of fecal microorganisms directly interacting with the HT29 cell surface were visualized (Figure S4). 

According to our data, in breast-fed infants the HT29 cell-associated GM fraction showed a phylogenetic structure at high taxonomic level that generally resembled the one observed in the fecal microbiota (Table 2). In particular, the HT29 cell-associated GM fraction of breast-fed infants was dominated by *Bifidobacterium* (rel. ab., 14%) and *Enterobacteriaceae* (35%) with *Bacteroides-Prevotella*, *Enterococcales*, *Clostridium* clusters IX and XIVa, *Lactobacillaceae* and *Bacillaceae* as minor members (11, 11, 5, 5, 7 and 6%, respectively). Respect to the fecal microbiota, the HT29 cell-associated fraction showed a trend (*FDR* < 0.3) towards the enrichment in *Enterobacteriaceae*, *Enterococcales*, *Bacillaceae*, *Lactobacillaceae*, and a corresponding reduction in *Clostridium* cluster IX. A significant enrichment in the minor (rel. ab. < 1%) microbiota component *Clostridium* cluster XI was also observed.

**Table 2:** High taxonomic level profile of the fecal microbiota and the HT29 cell-associated fraction in breast-fed infants and adults.

	**Breast-fed infants**			**Adults**		
**Microbial group**	**Fecal microbiota**	**HT29 cell-associated fraction**	***FRD***	**Fecal microbiota**	**HT29 cell-associated fraction**	***FDR***
***Bacteroides-Prevotella***	17.4	10.8	0.282	16.1	23.5	0.096
***Clostridium* cluster IV**	1.2	1.7	0.178	30.4	21.5	0.082
***Clostridium* cluster IX**	12.2	5.4	0.298	4.6	5.2	n.s.
***Clostridium* cluster XIVa**	5.9	5.0	n.s.	38.4	28.7	**0.006**
***Clostridium* cluster XI**	0.2	0.5	**0.014**	0.5	0.3	0.154
***Clostridium* cIuster I, II**	1.8	1.4	0.428	0.5	1.2	0.530
***Lactobacillaceae***	3.6	6.9	0.224	1.3	1.9	n.s.
***Bifidobacteriaceae***	24.1	14.4	n.s.	2.4	4.0	n.s.
***Verrucomicrobiae***	1.3	0.8	n.s.	1.3	3.3	n.s.
***Bacillaceae***	2.4	5.5	0.282	1.7	1.8	n.s.
***Fusobacteriaceae***	0.5	0.6	n.s.	1.0	1.1	n.s.
***Enterococcales***	4.3	10.9	0.428	0.9	1.6	0.406
***Enterobacteriaceae***	24.7	35.3	0.296	0.6	5.5	**0.004**
***Campylobacteriaceae***	0.4	0.7	**0.028**	0.4	0.3	n.s.

For each microbial group, mean relative abundance (%) and statistical significance (*FDR* < 0.05; bold) or tendency (*FDR* < 0.3) of the differences between the fecal microbiota and the HT29 cell-associated fraction are reported. Student’s t-test or Mann-Whitney U-test corrected for multiple comparison (*FDR*) were used. N.s., not significant.

Differently from breast-fed infants, the enterocyte-associated GM fraction in adults was remarkably different from the fecal one (Table 2). In particular, the HT29-cell associated fraction was significantly depleted in the dominant adult GM component *Clostridium* cluster XIVa, whose rel. ab. shifted from 38% to 29%. Moreover, concerning the other major GM members, a tendency towards the reduction of *Clostridium* cluster IV (rel. ab. from 30% to 21%) as well as the enrichment of *Bacteroides*-*Prevotella* (rel. ab. from 16% to 23%) was shown. Finally, *Enterobacteriaceae*, only a minor component of the fecal GM in adults, significantly increased to a rel. ab. of 5% in the HT29 cell-associated fraction. 

The comparison between the HT29 cell-associated GM fractions in breast-fed infants and adults revealed remarkable differences (Table 2), showing a HT29 cell-associated fraction dominated by *Bacteroides-Prevotella* and *Clostridium* clusters IV, IX, XIVa in adults, and by *Bifidobacterium* and *Enterobacteriaceae* in breast-fed infants.

### TNF-α differently modulates the HT29 cell-associated microbiota fraction in breast-fed infants and adults

HT29 cells have been reported to show an overall TNF-α response similar to the one proper of freshly isolated intestinal epithelial cells [45]. Thus, in order to investigate whether an inflammatory response modulated the mucosa-associated GM fraction, the interaction experiments between GM and HT29 cells were repeated with cell monolayers pre-treated with TNF-α (Figure S3). While the inflammatory stimulus resulted in a dramatic remodeling of the HT29 cell-associated GM fraction from adults, no changes were observed for infants (Table 3). In particular, in adults inflammation involved a strong and significant (FDR = 0.008) reduction of the dominant GM component *Bacteroides-Prevotella* from a rel. ab. of 23% in the absence of inflammatory stimuli to 15% in the presence of TNF-α. On the other hand, the TNF-α pre-treatment prompted a concomitant significant increase of the minor microbiota component *Bacillaceae*, whose rel. ab. in the HT29 cell-associated fraction of adults shifted from 2% in the absence of TNF-α to 4% in the presence of TNF-α. Further, a tendency (FDR = 0.154) towards the increase of the major GM component *Clostridium* cluster XIVa as a response to the inflammatory stimulus was observed. In order to exclude an intrinsic effect of TNF-α on the microbiota structure, fecal microbiota suspensions were incubated with or without TNF-α in the absence of HT29 cells, and subsequently characterized by means of the HTF-Microbi.Array/qPCR dual approach. According to our findings, community fingerprints of fecal suspensions in the presence or absence of TNF-α showed a Pearson’s correlation coefficient > 0.9, indicating the absence of any significant impact of TNF-α on the community composition (data not shown). 

**Table 3:** TNF-α impact on the HT29 cell-associated microbiota fraction in breast-fed infants and adults.

	**Breast-fed infants**			**Adults**		
**Microbial group**	**- TNF-α**	**+ TNF-α**	***FRD***	**- TNF-α**	**+ TNF-α**	***FRD***
***Bacteroides - Prevotella***	10.8	9.3	n.s.	23.5	15.4	**0.008**
***Clostridium* cluster IV**	1.7	2.5	0.520	21.5	18.2	n.s.
***Clostridium* cluster IX**	5.4	5.1	n.s.	5.2	3.4	n.s.
***Clostridium* cluster XIVa**	5.0	4.8	n.s.	28.7	37.5	0.154
***Clostridium* cluster XI**	0.5	0.6	n.s.	0.3	1.3	**<0.001**
***Clostridium* cIuster I, II**	1.4	1.3	n.s.	1.2	1.8	0.24
***Lactobacillaceae***	6.9	7.4	n.s.	1.9	2.1	n.s.
***Bifidobacteriaceae***	14.4	13.1	n.s.	4.0	5.8	n.s.
***Verrucomicrobiae***	0.8	1.0	n.s.	3.3	1.5	n.s.
***Bacillaceae***	5.5	6.3	n.s.	1.8	4.0	**0.004**
***Fusobacteriaceae***	0.6	0.8	0.570	1.1	1.7	0.132
***Enterococcales***	10.9	10.4	n.s.	1.6	2.5	0.430
***Enterobacteriaceae***	35.3	36.8	n.s.	5.5	3.8	0.360
***Campylobacteriaceae***	0.7	0.8	n.s.	0.3	1.1	**<0.001**

For each microbial group, mean relative abundance (%) and statistical significance (*FDR* < 0.05; bold) or tendency (*FDR* < 0.3) of the differences between the fecal microbiota and the HT29 cell-associated fraction are reported. Student’s t-test or Mann-Whitney U-test corrected for multiple comparison (*FDR*) were used. N.s., not significant.

## Discussion

The comparative 16S rDNA pyrosequencing study of the GM of breast-fed infants and adults demonstrated profound structural differences between the two ecosystems. Respect to adults, the breast-fed infant GM was characterized by a lower compositional diversity and a higher degree of interindividual variability. The adult GM was largely dominated by *Bacteroidetes* and *Firmicutes*, whereas the GM of breast-fed infants showed *Bifidobacterium* as a predominant component with a lower proportion of *Clostridiales*. Moreover, compared to adults, the GM of breast-fed infants was largely enriched in facultatively aerobic populations, such as *Streptococcaceae*, *Enterococcaceae* and *Enterobacteriaceae*.

We previously developed a HTF-Microbi.Array/qPCR combined approach for the high taxonomic level profiling of the GM structure, which has been proved to be a robust and rapid tool to characterize the gut microbial communities in infants and adults [28]. Focusing its phylogenetic resolution to the level of order and cluster, the HTF-Microbi.Array is blind with respect to the astonishing interindividual variability of GM at the species level, being specifically conceived to characterize different GM phylogenetic assets with the potential to differently modulate host physiology [27,46]. In the present paper, through a Procrustes similarity analysis of the HTF-Microbi.Array/qPCR-based fingerprints of the fecal microbial communities in breast-fed infants and adults, and the ones obtained by 16S rDNA pyrosequencing, we showed a significant agreement between the two approaches, further supporting the reliability of our phylogenetic array platform for the high taxonomic level fingerprinting of the human GM.

In order to shed some light on the peculiarity of the interplay between GM and the host immune system at different ages, we developed a non-invasive minimal model based on the interaction between the human enterocyte line HT29 and microbial community suspensions obtained from fresh stools. The enterocyte-associated GM fractions from breast-fed infants and adults were compared by means of the HTF-Microbi.Array/qPCR combined approach. The mucus-secreting HT29 cells were selected since considered among the most relevant enterocyte cell lines available for *in vitro* reproducing physiology and immune function of the human intestinal mucosa [26,47]. Moreover, remaining undifferentiated to 95% in the post-confluent state, HT29 cells have been recently indicated as a suitable *in vitro* model for reproducing the immature intestinal environment of a neonate [48]. 

Our *ex vivo* model reflects remarkable differences between the enterocyte-associated GM fractions in breast-fed infants and adults. While the enterocyte-associated microbiota in breast-fed infants showed *Bifidobacterium* and *Enterobacteriaceae* as dominant components - with *Bacteroides-Prevotella*, *Enterococcales, Clostridium* clusters IX and XIVa, *Lactobacillaceae* and *Bacillaceae* as subdominant - in adults, the enterocyte adherent GM fraction was largely dominated by *Bacteroides-Prevotella* and *Clostridium* clusters IV and XIVa, with *Clostridium* cluster XI, *Enterobacteriaceae* and *Bifidobacterium* as minor components. These data are in general agreement with that observed by Durban et al. [49] and Ouwehand et al. [50] in bioptic samples collected from healthy adults and infants, respectively. While in infants, the phylogenetic structure of the enterocyte-associated GM fraction generally resembled the one observed in the fecal microbiota, in adults it showed a totally different structure, being enriched in *Bacteroides-Prevotella* and *Enterobacteriaceae* and depleted in *Clostridium* clusters IV and XIVa. This observation suggests that the GM of breast-fed infants is selected for a closer interaction with the host enterocytes, a factor that may be important for the establishment of the intense cross-talk with the host immune system which drives the process of immune education [51]. In fact, enriched in *Bifidobacterium* and *Enterobacteriaceae*, the infant-type enterocyte-associated microbiota fraction is specifically structured to drive immune education in early infancy [52]. While *Enterobacteriaceae* provide genes associated with virulence functionality, which continuously boost the immunological response [53], the early bifidobacterial fraction - dominated by the species *B. breve* and *B. longum* [13,54] - has been shown to exert synergic immune modulatory and protective properties [55,56], allowing the promotion and maintenance of a mutualistic cross-talk between the infant immune system and the mucosa-associated GM fraction [11]. 

Equipped with a vast array of Toll-like receptors, enterocytes can sense intestinal microorganisms, resulting in the recruitment and activation of macrophages and dendritic cells. This leads to the secretion of TNF-α and other pro-inflammatory cytokines, establishing a host pro-inflammatory response [11,23,57]. In the light of this, in order to assess the impact of inflammation on the microbial ecology of the microbiota-enterocyte interaction process, HT29 cell interaction experiments were repeated with TNF-α. In particular, changing the expression of enterocyte surface proteins, pro-inflammatory cytokines can modify the enterocyte microenvironment [58]. For instance, it has been reported that in response to TNF-α enterocytes up-regulate the expression of polymeric Ig receptor [59], a non-specific microbial scavenger which limits the bacterial interaction with the enterocyte surface [60]. According to our data, the enterocyte response to the TNF-α-dependent pro-inflammatory stimulus is sufficient to compromise the enterocyte-associated GM community in adults, while it does not impair the enterocyte adherent GM of breast-fed infants, demonstrating that this microbial community structure is structured to cope with host inflammatory responses. Taken together, our findings suggest that infant- and adult-type microbiota possess different functional properties for what concerns the capacity to interact with enterocytes, suggesting the specificity of the interaction process between GM and the host immune system at different ages. Indeed, the infant-type microbiota has co-evolved to cope with the process of immune education, which is based on transient inflammatory signals from gut microorganisms, functional to prime the early immune response [11,51]. Differently, the adult-type microbiota is structured to forge and preserve a homeostatic low-grade inflammatory status, and is dramatically compromised by host inflammatory responses [61]. 

In conclusion, employing our reductionist non-invasive *ex vivo* HT29 cell-based model, we demonstrated that the infant- and adult-type enterocyte-associated GM fractions possess different phylogenetic and functional structures strategic to respond to the specific immunological needs at different ages. Our findings open the perspective to the usage of our *ex vivo* system in non-invasive case-control screening studies aimed at characterizing disease-associated deviations from a healthy enterocyte-associated GM profile, either in adults or infants. These studies could disclose specific dysbioses in the mucosa-associated microbiota fraction that – as the first line in the interaction with the host immune system – could potentially be involved in emerging inflammatory diseases, such as allergy, inflammatory bowel disease, inflammatory bowel syndrome and obesity-associated metabolic endotoxemia. 

## Supporting Information

Figure S1 **Relative abundance of phylum (**a**)- and genus (**b**)-classified fecal microbiota in breast-fed infants (left side) and adults (right side).** Histograms are based on the proportion of OTUs per subject. Colors were assigned for all phyla detected (a), and for genera with a relative abundance ≥ 1% in at least 20% of subjects (b).
(JPG)

Figure S2 **High taxomonic level fingerprint of the fecal microbiota of breast-fed infants (blue) and adults (red) using HTF-Microbi.Array/qPCR combined methodology correlates with pyrosequencing data.** Procrustes analysis combining weighted UniFrac PCoA from pyrosequencing (non-circle end of lines) with PCA from HTF-Microbi.Array/qPCR data (circle end of lines) is shown.
(JPG)

Figure S3 **High taxonomic level profile of the fecal microbiota and HT29 cell-associated fraction in breast-fed infants and adults.** Pie charts of the mean values of relative abundance (%) of the major microbial groups in the fecal microbiota (Slurry) and the HT29 cell-associated fraction in the absence (Adh) or presence (TNF-α) of a TNF-α-mediated pro-inflammatory stimulus, in breast-fed infants (left panel) and adults (right panel). The relative contribution of each microbial group was determined using the HTF-Microbi.Array/qPCR combined methodology. The HT29 cell-associated fraction was evaluated after 1-h interaction of fecal slurries with HT29 cell monolayers, previously stimulated or not with 2 ng/ml of human TNF-α for 24 h.
(JPG)

Figure S4 **Visualization of the HT29 cell-associated microbiota fraction.** Fecal bacteria were stained with DAPI and allowed to interact with HT29 cell monolayers grown on glass coverslips. After fixing, samples were visualized under both fluorescent light and phase contrast. For each experimental condition, the assay was repeated 3 times. Representative images for an adult (A) and a breast-fed infant (B) are shown (magnification, 100x). Left panel, DAPI-stained adherent bacteria (blue) under fluorescent light. Right panel, corresponding phase contrast images showing the underlying HT29 cell layer.
(JPG)

Table S1 **Age of each subject enrolled in the study.**
(XLSX)
