# Supplementary figures and images for: The Enterocyte-Associated Intestinal Microbiota of Breast-Fed Infants and Adults Responds Differently to a TNF-α-Mediated Pro-Inflammatory Stimulus
Source: PLoS One. 2013 Nov 26;8(11):e81762. doi: 10.1371/journal.pone.0081762 (PMC3841132; doi:10.1371/journal.pone.0081762)

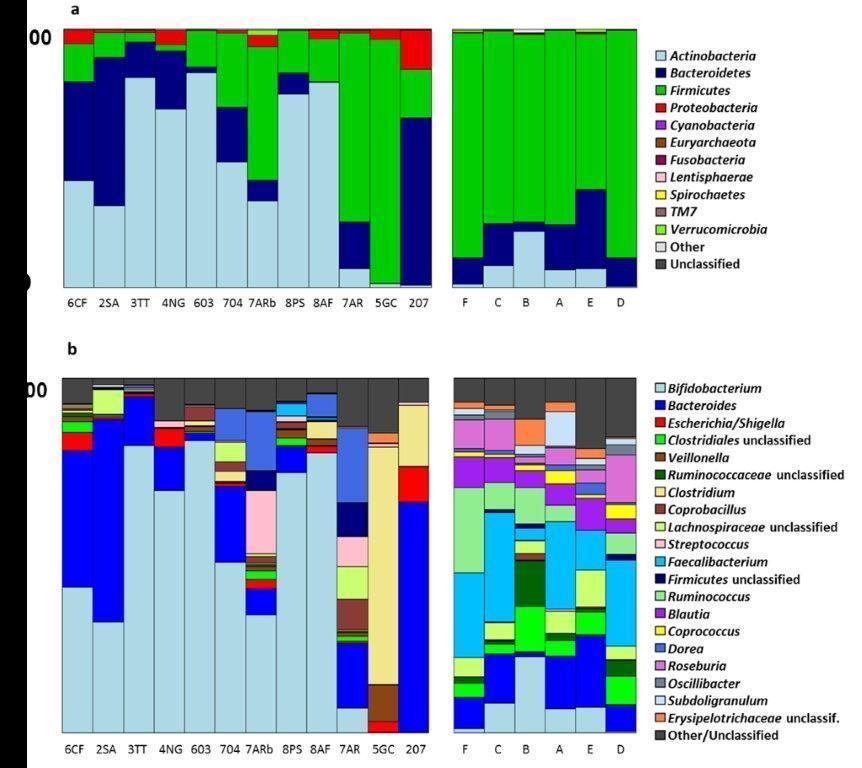

Supplement: Figure S1 — Relative abundance of phylum (a)- and genus (b)-classified fecal microbiota in breast-fed infants (left side) and adults (right side). Histograms are based on the proportion of OTUs per subject. Colors were assigned for all phyla detected (a), and for genera with a relative abundance ≥ 1% in at least 20% of subjects (b). (JPG) [file pone.0081762.s001.jpg]

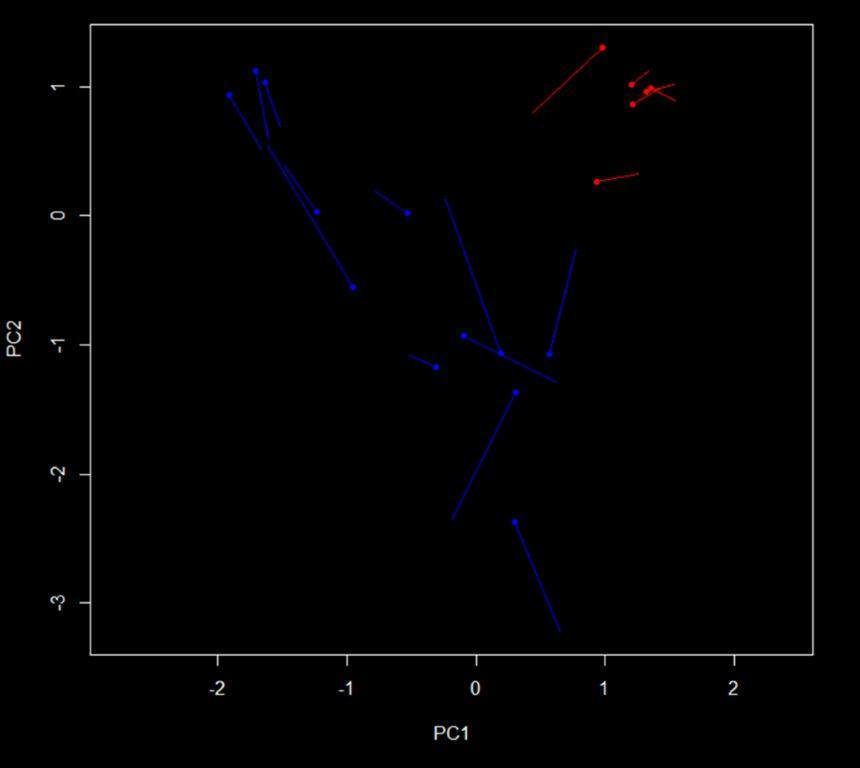

Supplement: Figure S2 — High taxomonic level fingerprint of the fecal microbiota of breast-fed infants (blue) and adults (red) using HTF-Microbi.Array/qPCR combined methodology correlates with pyrosequencing data. Procrustes analysis combining weighted UniFrac PCoA from pyrosequencing (non-circle end of lines) with PCA from HTF-Microbi.Array/qPCR data (circle end of lines) is shown. (JPG) [file pone.0081762.s002.jpg]

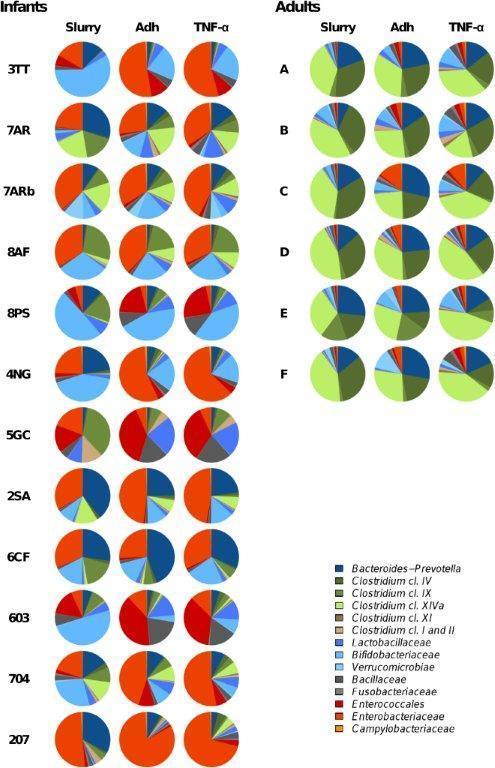

Supplement: Figure S3 — High taxonomic level profile of the fecal microbiota and HT29 cell-associated fraction in breast-fed infants and adults. Pie charts of the mean values of relative abundance (%) of the major microbial groups in the fecal microbiota (Slurry) and the HT29 cell-associated fraction in the absence (Adh) or presence (TNF-α) of a TNF-α-mediated pro-inflammatory stimulus, in breast-fed infants (left panel) and adults (right panel). The relative contribution of each microbial group was determined using the HTF-Microbi.Array/qPCR combined methodology. The HT29 cell-associated fraction was evaluated after 1-h interaction of fecal slurries with HT29 cell monolayers, previously stimulated or not with 2 ng/ml of human TNF-α for 24 h. (JPG) [file pone.0081762.s003.jpg]

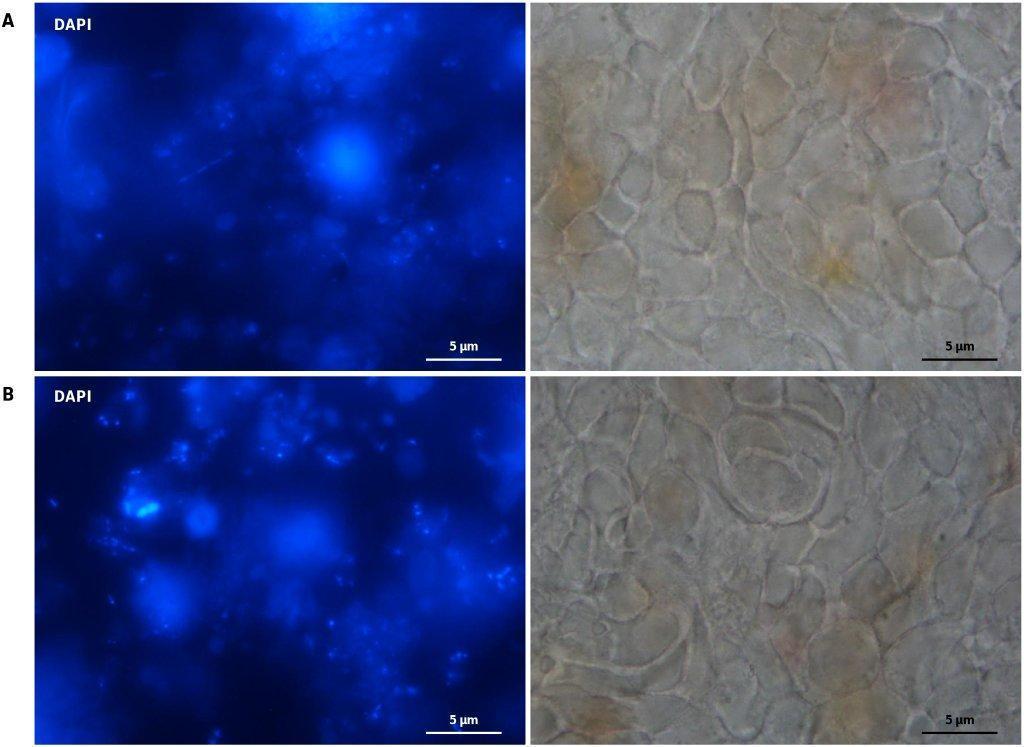

Supplement: Figure S4 — Visualization of the HT29 cell-associated microbiota fraction. Fecal bacteria were stained with DAPI and allowed to interact with HT29 cell monolayers grown on glass coverslips. After fixing, samples were visualized under both fluorescent light and phase contrast. For each experimental condition, the assay was repeated 3 times. Representative images for an adult (A) and a breast-fed infant (B) are shown (magnification, 100x). Left panel, DAPI-stained adherent bacteria (blue) under fluorescent light. Right panel, corresponding phase contrast images showing the underlying HT29 cell layer. (JPG) [file pone.0081762.s004.jpg]
